# The biochemistry of acetaminophen hepatotoxicity and rescue: a mathematical model

**DOI:** 10.1186/1742-4682-9-55

**Published:** 2012-12-19

**Authors:** Rotem Ben-Shachar, Yifei Chen, Shishi Luo, Catherine Hartman, Michael Reed, H Frederik Nijhout

**Affiliations:** 1 Program in Computational Biology and Bioinformatics, Duke University, Durham, NC, USA; 2 Program in Biophysics, Duke University, Durham, NC, USA; 3 Department of Mathematics, Duke University, Durham, NC, USA; 4 Department of Biology, Duke University, Durham, NC, USA

**Keywords:** Acetaminophen, Hepatotoxicity, Mathematical model, Glutathione, NAPQI

## Abstract

**Background:**

Acetaminophen (N-acetyl-para-aminophenol) is the most widely used over-the-counter or prescription painkiller in the world. Acetaminophen is metabolized in the liver where a toxic byproduct is produced that can be removed by conjugation with glutathione. Acetaminophen overdoses, either accidental or intentional, are the leading cause of acute liver failure in the United States, accounting for 56,000 emergency room visits per year. The standard treatment for overdose is N-acetyl-cysteine (NAC), which is given to stimulate the production of glutathione.

**Methods:**

We have created a mathematical model for acetaminophen transport and metabolism including the following compartments: gut, plasma, liver, tissue, urine. In the liver compartment the metabolism of acetaminophen includes sulfation, glucoronidation, conjugation with glutathione, production of the toxic metabolite, and liver damage, taking biochemical parameters from the literature whenever possible. This model is then connected to a previously constructed model of glutathione metabolism.

**Results:**

We show that our model accurately reproduces published clinical and experimental data on the dose-dependent time course of acetaminophen in the plasma, the accumulation of acetaminophen and its metabolites in the urine, and the depletion of glutathione caused by conjugation with the toxic product. We use the model to study the extent of liver damage caused by overdoses or by chronic use of therapeutic doses, and the effects of polymorphisms in glucoronidation enzymes. We use the model to study the depletion of glutathione and the effect of the size and timing of N-acetyl-cysteine doses given as an antidote. Our model accurately predicts patient death or recovery depending on size of APAP overdose and time of treatment.

**Conclusions:**

The mathematical model provides a new tool for studying the effects of various doses of acetaminophen on the liver metabolism of acetaminophen and glutathione. It can be used to study how the metabolism of acetaminophen depends on the expression level of liver enzymes. Finally, it can be used to predict patient metabolic and physiological responses to APAP doses and different NAC dosing strategies.

## Background

Acetaminophen (N-acetyl-para-aminophenol, APAP or paracetamol) is the most widely used over-the- counter and prescription painkiller in the world [1]. While safe at therapeutic doses of up to 4 grams per day for adults, acetaminophen overdoses, either accidental or intentional, are the leading cause of acute liver failure in the United States, accounting for some 56,000 emergency room visits, 2,600 hospitalizations and nearly 500 deaths annually [2,3].

**Figure 1:**
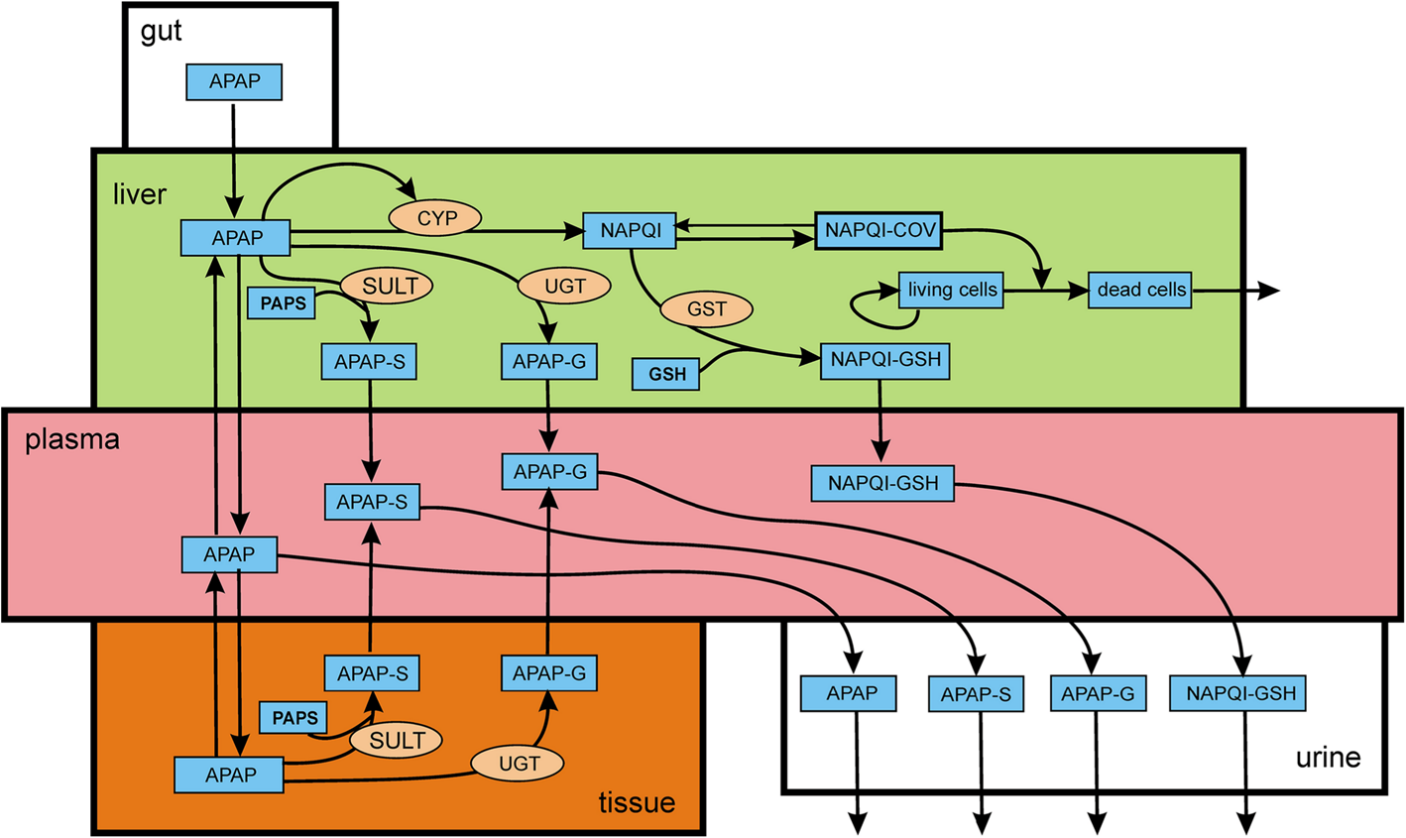
**Acetaminophen metabolism.** Blue boxes indicate substrates: APAP, acetaminophen; APAP-S, APAP-sulfonate; APAP-G, APAP-glucoronidate; NAPQI, N-acetyl-p-benzoquinone imine; NAPQI-COV, covalent binding of NAPQI; NAPQI-GSH, NAPQI conjugated with glutathione; PAPS, 3’-Phosphoadenosine-5’-phosphosulfate; GSH, glutathione. The light orange ovals indicate the enzymes that catalyze reactions: SULT, sulfotransferase; UGT, glucuronosyltransferase; CYP, cytochrome P-450 oxidase; GST, glutathione S-transferase.

Acetaminophen is metabolized by conjugation with sulfate and glucoronidate, which are inert and are excreted in the urine. Depending on dose, a fraction of APAP is converted into a highly reactive toxic intermediate, N-acetyl-p-benzoquinone imine (NAPQI) by several P450 cytochromes [4]. Substantial amounts of NAPQI are effectively eliminated by conjugation with glutathione (GSH). However, after a large dose of APAP, the sulfonation reaction becomes saturated and the build up of NAPQI depletes GSH in the liver, causing further accumulation of NAPQI. Unconjugated NAPQI binds to proteins and subcellular structures and induces rapid cell death and necrosis that can lead to liver failure. The main biochemical pathways of acetaminophen metabolism and the transports between various compartments are pictured in Figure 1.

N-acetylcysteine (NAC) can be an effective antidote for APAP poisoning. NAC limits hepatotoxicity by increasing GSH synthesis in the liver [5]. Current protocols recommend treating patients with an initial dose of 150 mg/kg NAC, infused over a period of an hour, upon hospitalization, followed by decreasing amounts of NAC infused over the next 20 hours [1]. Fatal liver damage can be prevented if the initial dose of NAC is administered within 8-12 hours of an APAP overdose. This antidote dosage regime has been developed empirically over a period of many years based on outcomes from clinical cases. It is not known whether the current NAC treatment protocol is optimal.

The metabolism of APAP has been well-studied and the distributions of its metabolites in the plasma and urine of humans are well-documented [4,6,7], as are the hepatic values in mice and rats [8]. What has been lacking is an integrated and quantitative understanding of the kinetics of APAP metabolism, of how APAP dosage affects NAPQI synthesis and GSH concentrations in the liver, of how NAC stimulates the synthesis of GSH, and of how the dosage and timing of NAC affect detoxification of NAPQI. In this paper we develop a mathematical model for APAP metabolism that allows us to study, *in silico*, how various doses of APAP are metabolized and whether or not a dose exceeds the capacity of the liver to synthesize sufficient GSH. In order to study how the metabolism of APAP affects GSH concentration and resynthesis, we have connected the model depicted in Figure 1 to our extant model of glutathione metabolism [9]. This enables us to examine the effect of GSH synthesis capacity on the ability of hepatocytes to detoxify NAPQI, the accumulation of NAPQI-induced liver damage, and the effects of different doses and timing of NAC in emergency departments.

Remien et al. [10] recently developed a mathematical model to estimate overdosage of APAP based on indicators of liver damage (blood levels of aspartate aminotransferase, alanine aminotransferase and the international normalized ratio of prothrombin time) that are measured upon admission to hospital emergency departments. In a retrospective study, this model was able to accurately predict whether the overdose would lead to fatal liver damage. Our model is complementary to the work of [10] since it focuses on the detailed biochemical mechanisms by which of APAP is detoxified in the liver under both normal and overdose situations.

## Methods

The mathematical model consists of 21 differential equations for the variables listed in Table 1. The differential equations corresponding to the reactions diagramed in Figure 1 are listed below. Lower case *p*,*l*,*t*, and *u* refer to plasma, liver, tissue and urine respectively. We use lower case italic abbreviations in the differential equations and other formulas so that they are easy to read and are not confused with enzyme names which are in caps. Full names for the enzymes appear in the legend to Figure 1. Reaction velocities or transport velocities begin with a capital V followed by the name of the enzyme, the transporter, or the process as a subscript. For example, *V*_lSULT_(*lapap*,*lpaps*) is the velocity of the sulfation reaction in the liver, which depends on the concentrations of the substrates, *lapap* and *lpaps*. After the differential equations, we discuss in detail the more difficult modeling issues and reactions with non-standard kinetics. Table 2 gives the assumed values of volumes, transport parameters, and hepatocyte parameters. Table 3 gives the parameter choices and references for biochemical reactions.

**Table 1:** Names used for Variables

**In equations**	**In text**	**Full name**
*gapap*	gAPAP	APAP in the gut
*papap*	pAPAP	APAP in the plasma
*pas*	pAPAP-S	APAP sulfonate in the plasma
*pag*	pAPAP-G	APAP glucoronidate in the plasma
*pnqgsh*	pNAPQI-GSH	NAPQI-GSH complex in the plasma
*lapap*	lAPAP	APAP in the liver
*lpaps*	lPAPS	liver phosphoadenosine-phosphosulfate
*las*	lAPAP-S	APAP sulfonate in the liver
*lag*	lAPAP-G	APAP glucoronidate in the liver
*lnq*	lNAPQI	NAPQI in the liver
*lcov*	covalent binding	covalent binding of NAPQI in the liver
*lnqgsh*	lNAPQI-GSH	NAPQI-GSH complex in the liver
*lgsh*	lGSH	GSH in the liver
*lh*	lH	functional hepatocytes
*lz*	lZ	damaged hepatocytes
*tapap*	tAPAP	APAP in the tissue
*tpaps*	tPAPS	tissue phosphoadenosine-phosphosulfate
*tas*	tAPAP-S	APAP sulfonate in the tissue
*tag*	tAPAP-G	APAP glucoronidate in the tissue
*uapap*	uAPAP	APAP in the urine
*uas*	uAPAP-S	APAP sulfonate in the urine
*uag*	uAPAP-G	APAP glucoronidate in the urine
*unq*	uNAPQI	NAPQI in the urine
*unqgsh*	uNAPQI-GSH	NAPQI-GSH complex in the urine

**Table 2:** **Parameters (*μ*M,*μ*M/hr,/hr)**

**Parameter**	**Value**	**Parameter Description**
Compartment volumes (liters)		
*vG*	1	gut
*vP*	3	plasma
*vL*	1.5	liver
*vT*	30	tissue
*vU*	1.5	urine
Transport parameters (*μ*M/hr)		
*k*_*gl*_	4	APAP from gut to liver
*k*_*pt*_	0.39	APAP from plasma to tissue
*k*_*tp*_	0.78	APAP from tissue to plasma
*k*_*pl*_	0.0225	APAP from plasma to liver
*k*_*lp*_	0.2025	APAP from liver to plasma
*k*_*slp*_	0.24	sulfonate from liver to plasma
*k*_*stp*_	0.0913	sulfonate from tissue to plasma
*k*_*glp*_	0.3024	glucoronidate from liver to plasma
*k*_*gtp*_	0.5171	glucoronidate from tissue to plasma
*k*_*nqglp*_	0.3	NAPQI-GSH conjugate from liver to plasma
*k*_*pu*_	0.06	APAP from plasma to urine
*k*_*spu*_	4	sulfonate from plasma to urine
*k*_*gpu*_	3.8	glucoronidate from pasma to urine
*k*_*nqgpu*_	0.4	NAPQI-GSH conjugate from plasma to urine
*k*_*u*_	1.54	urine elimination
Hepatocyte parameters		
*r*	.0417	logistic growth (*hr*^−1^)
*h*_*max*_	(1*.*6)10^11^	maximum hepatocyte number
*η*	(*.*213)10^−4*.*2^	cell death rate due to NAPQI (*hr*^−1^*μM*^−1^)
*δ*_*z*_	.2083	removal of dead hepatocytes (*hr*^−1^)

**Table 3:** **Biochemical Parameters (*μ*M,*μ*M/hr,/hr)**

**Reaction**	**Parameter**	**Model value**	**Literature value**	**References**	
*V*_*CYP*_					
	p	20			
	d	18000			
	n	2			
*V*_CYP1A2_	cytochrome CYP1A2				
	*K*_*m*_	3430	3430-3440	[16]	
	*V*_*max*_	0.55			
*V*_CYP2E1_	cytochrome CYP2E1				
	*K*_*m*_	677	677-1260	[16]	
	*V*_*max*_	345			
*V*_CYP3A4_	cytochrome CYP3A4				
	*K*_*m*_	276	276-313	[16]	
	*V*_*max*_	0.99			
*V*_(l/t)SULT_	sulfation				
	$\left(K_{m}^{\text{apap}}\right)$	97	97	[24]	
	$\left(K_{m}^{\text{paps}}\right)$	5.6	5.6	[25]	
	*V*_*max*_ in liver	1785			
	*V*_*max*_ in tissue	357			
PAPS metabolism					
	*V*_*lpaps*_ (synthesis liver)	0.1			
	*k*_*lpaps*_ (linear removal)	0.0033			
	*V*_*tpaps*_ (synthesis tissue)	0.01			
	*k*_*tpaps*_ (linear removal)	0.00033			
*V*_GST_	glutathione transferase				
	$\left(K_{m}^{\text{GSH}}\right)$	5200	4600–5600	[21,22]	
	$\left(K_{m}^{\text{NAPQI}}\right)$	15			
	*V*_*max*_	72000			
covalent binding					
	*k*_*bind*_	1000			
	*k*_*rev*_	0.25			
*V*_(l/t)UGT_=*V*_UGT1_ + *V*_UGT2_ + *V*_UGT3_ + *V*_UGT4_					
*V*_UGT1_	glucoronidation				
	*K*_*m*_	5500	5500	[20]	
	*n* (Hill)	3.2			
	*V*_*max*_ in liver	6370			
	*V*_*max*_ in tissue	1274			
*V*_UGT2_	glucoronidation				
	*K*_*m*_	4000	4000	[20]	
	*K*_*i*_	23000	23000	[20]	
	*V*_*max*_ in liver	490			
	*V*_*max*_ in tissue	98			
*V*_UGT3_	glucoronidation				
	*K*_*m*_	9200	9200	[20]	
	*V*_*max*_ in liver	4900			
	*V*_*max*_ in tissue	980			
*V*_UGT4_	glucoronidation				
	*K*_*m*_	23000	23000	[20]	
	*K*_*i*_	5300	5300	[20]	
	*V*_*max*_ in liver	7350			
	*V*_*max*_ in tissue	1470			

The differential equations for the variables listed in Table 1 are:

$$\begin{array}{l} \;\frac{d[\text{gapap}]}{\text{dt}}=-k_{\text{gl}}\cdot \text{gapap}\\ \;\frac{d[\text{papap}]}{\text{dt}}=k_{\text{tp}}\cdot (\text{vT}/\text{vP})\cdot \text{tapap}\;-\;k_{\text{pt}}\cdot \text{papap}\;+\;k_{\text{lp}}\cdot (\text{vL}/\text{vP})\cdot \text{lapap}-k_{\text{pu}}\cdot \text{papap}\\ \;\;\frac{d[\text{pas}]}{\text{dt}}=k_{\text{slp}}\cdot (\text{vL}/\text{vP})\cdot \text{las}+k_{\text{stp}}\cdot (\text{vT}/\text{vP})\cdot \text{tas}-k_{\text{spu}}\cdot \text{pas}\\ \;\;\frac{d[\text{pag}]}{\text{dt}}=k_{\text{glp}}\cdot (\text{vL}/\text{vP})\cdot \text{lag}+k_{\text{gtp}}\cdot (\text{vT}/\text{vP})\cdot \text{tag}-k_{\text{gpu}}\cdot \text{pag}\\ \frac{d[\text{pnqgsh}]}{\text{dt}}=k_{\text{nqglp}}\cdot (\text{vL}/\text{vP})\cdot \text{lnqgsh}-k_{\text{nqgpu}}\cdot \text{pnqgsh}\\ \;\frac{d[\text{lapap}]}{\text{dt}}=k_{\text{gl}}\cdot (\text{vG}/\text{vL})\cdot \text{gapap}+k_{\text{pl}}\cdot (\text{vP}/\text{vL})\cdot \text{papap}-V_{\text{CYP}}(\text{lapap})\\ \;\;\;\;\;-V_{\text{lSULT}}(\text{lapap},\text{lpaps})-V_{\text{lUGT}}(\text{lapap})\\ \;\;\frac{d[\text{las}]}{\text{dt}}=V_{\text{lSULT}}(\text{lapap},\text{lpaps})-k_{\text{slp}}\cdot \text{las}\\ \;\;\frac{d[\text{lag}]}{\text{dt}}=V_{\text{lUGT}}(\text{lapap})-k_{\text{glp}}\cdot \text{lag}\\ \;\;\frac{d[\text{lnq}]}{\text{dt}}=V_{\text{CYP}}(\text{lapap})-V_{\text{GST}}(\text{lnq},\text{lgsh})+k_{\text{rev}}\cdot \text{lcov}-k_{\text{bind}}\cdot \text{lnq}\\ \;\frac{d[\text{lcov}]}{\text{dt}}=k_{\text{bind}}\cdot \text{lnq}\;-k_{\text{rev}}\cdot \text{lcov}\\ \;\;\frac{d[\text{lh}]}{\text{dt}}=r\cdot \text{lh}\cdot (1-\frac{\left(\text{lh}+\text{lz}\right)}{h_{\text{max}}})-\eta \cdot \text{lcov}\cdot \text{lh}\\ \;\;\frac{d[\text{lz}]}{\text{dt}}=\eta \cdot \text{lcov}\cdot \text{lh}-\delta_{z}\cdot \text{lz}\\ \;\frac{d[\text{lpaps}]}{\text{dt}}=V_{\text{lpaps}}-V_{\text{lSULT}}(\text{lapap},\text{lpaps})-k_{\text{lpaps}}\cdot \text{lpaps}\\ \;\frac{d[\text{tapap}]}{\text{dt}}=k_{\text{pt}}\cdot (\text{vB}/\text{vT})\cdot \text{papap}-k_{\text{tp}}\cdot \text{tapap}-V_{\text{tSULT}}(\text{tapap},\text{tpaps})\\ \;\;\;\;\;-V_{\text{tUGT}}(\text{tapap})\\ \;\;\frac{d[\text{tas}]}{\text{dt}}=V_{\text{tSULT}}(\text{tapap},\text{tpaps})-k_{\text{stp}}\cdot \text{tas}\\ \;\;\frac{d[\text{tag}]}{\text{dt}}=V_{\text{tUGT}}(\text{tapap})-k_{\text{gtp}}\cdot \text{tag}\\ \;\frac{d[\text{tpaps}]}{\text{dt}}=V_{\text{tpaps}}-V_{\text{tSULT}}(\text{tapap},\text{tpaps})-k_{\text{tpaps}}\cdot \text{tpaps}\\ \;\frac{d[\text{uapap}]}{\text{dt}}=k_{\text{pu}}\cdot (\text{vB}/\text{vU})\cdot \text{papap}-k_{u}\cdot \text{uapap}\\ \;\;\frac{d[\text{uas}]}{\text{dt}}=k_{\text{spu}}\cdot (\text{vB}/\text{vU})\cdot \text{pas}-k_{u}\cdot \text{uas}\\ \;\;\frac{d[\text{uag}]}{\text{dt}}=k_{\text{gpu}}\cdot (\text{vB}/\text{vU})\cdot \text{pag}-k_{u}\cdot \text{uag}\\ \frac{d[\text{unqgsh}]}{\text{dt}}=k_{\text{nqgpu}}\cdot \text{pnqgsh}-k_{u}\cdot \text{unqgsh} \end{array}$$

### Absorption and dosing

APAP is absorbed from the gut into the portal circulation which flows into the liver. In our model, our oral doses are deposited in the gut compartment and then removed and put into the liver with linear kinetics. In [11] the half life for gastric emptying was calculated to be 7 minutes for oral liquid doses and overnight fasting. We take the linear rate constant to be 4*μ*M/hr, which gives a half-life of approximately 10 minutes, since absorption will be slower with pills and non-fasted state. Most of the dose is absorbed in 30 to 60 minutes. The bioavailability of APAP is known to vary considerably depending on age, method of administration, and gut contents. An early study [12] measured an average bioavailability of 79% and a recent study [13] found a range 63%–89%. We assume that the bioavailability of a dose is 75%. A standard therapeutic dose is variously reported as 1000 mg or 20 mg/kg. In our model, we assume a 60 kg individual and a dose of 20mg/kg, which would make that standard dose 1200 mg. To convert those values to molarity in the gut we assumed a gut volume of 1 liter; then a 20 mg/kg dose (the typical therapeutic dose) produces a gut concentration of 6000 *μ*M, assuming 75% bioavailability.

### Cytochrome oxidase

Many P450 enzymes catalyze the production of NAPQI from APAP [14,15]. In our model, NAPQI is produced in the liver by three cytochrome oxidases, CYP2E1, CYP3A4, and CYP1A2. We assume each is Michaelis-Menten and take the *K*_*m*_values from [16]. Cyp3A4 dominates by having a much larger *V*_*max*_ than the other two enzymes.

$$\;V_{\text{CYP}}\;=\;\left(V_{\text{CYP1A2}}(\text{lapap})\;+\;V_{\text{CYP2E1}}(\text{lapap})\;+\;V_{\text{CYP3A4}}(\text{lapap})\right)\left(1+\frac{p\cdot {\left(\text{lapap}\right)}^{n}}{d^{n}+{\left(\text{lapap}\right)}^{n}}\right)$$

Allosteric activation, including substrate activation, of P450 enzymes has been extensively documented [17-19]. We have included substrate activation (the Hill term on the right) and found that if we omitted this substrate activation then the cytochrome oxidase reactions did not produce enough NAPQI at high overdoses.

### Glucoronidation

There are four glucoronosyltransferases, UGT 2B15, UGT 1A1, UGT 1A6, UGT 1A9 (that we denote simply by UGT1, UGT2, UGT3, and UGT4), that glucoronidate APAP. Each has somewhat different kinetics with different parameters: UGT3 has simple Michaelis-Menten kinetics; UGT1 has Hill kinetics; UGT2 and UGT4 show substrate inhibition [20].

$$\begin{array}{l} V_{\text{UGT1}}=\frac{V_{\text{max}1}{\left(\text{lapap}\right)}^{n}}{{\left(K_{m1}\right)}^{n}+{\left(\text{lapap}\right)}^{n}}\\ V_{\text{UGT2}}=\frac{V_{\text{max}2}(\text{lapap})}{K_{m2}+(\text{lapap})(1+\frac{\text{lapap}}{K_{i2}})}\\ V_{\text{UGT3}}=\frac{V_{\text{max}3}(\text{lapap})}{K_{m3}+(\text{lapap})}\\ V_{\text{UGT4}}=\frac{V_{\text{max}4}(\text{lapap})}{K_{m4}+(\text{lapap})(1+\frac{\text{lapap}}{K_{i4}})} \end{array}$$

### Glutathione synthesis and metabolism

We use a previously published model of liver glutathione metabolism [9]. That model is connected to the model for APAP metabolism described here by adding the reaction by which GSH conjugates NAPQI via the enzyme GST. This enables us to study how different doses of APAP decrease liver GSH and how that affects the formation of NAPQI. We take the *K*_*m*_ of GST for GSH to be 5200 *μ*M, midway between the values 4500 [21] and 5600 [22], *K*_*m *_= 15*μ*M for NAPQI, and *V*_*max *_= 72,000*μ*M/hr.

### Sulfation

APAP can be detoxified by being sulfated in a reaction with PAPS [23]. We take the reaction to have standard bi-bi kinetics with *K*_*m *_= 97*μ*M for APAP [24] and *K*_*m *_= 5*.*6*μ*M for PAPS [25].

### Covalent binding

NAPQI is believed to exert its toxic effects by binding covalently to liver proteins leading to protein denaturation and necrosis of liver cells [6,26,27]. We model the reaction as linear and reversible because covalent binding of NAPQI gradually declines after eight hours [28,29].

### Hepatic necrosis

The rate at which functional hepatocytes are damaged is proportional to the product of the number of functional hepatocytes and the concentration of covalent binding of NAPQI. We use the differential equations for the rate of change of the number of living hepatic cells and the rate of change of the number of damaged cells from [10].

### Transport

There are few measurements of overall transport rates of the metabolites between the compartments of the model. We chose to make all transport rates linear and adjusted them so that the measured APAP, APAP-S, APAP-G, and NAPQI-GSH concentrations in the plasma and the urine were as measured in the literature [4,6,11,30-32]. Values of the transport coefficients are given in Table 2.

## Results

### Model comparison to experimental data

**Figure 2:**
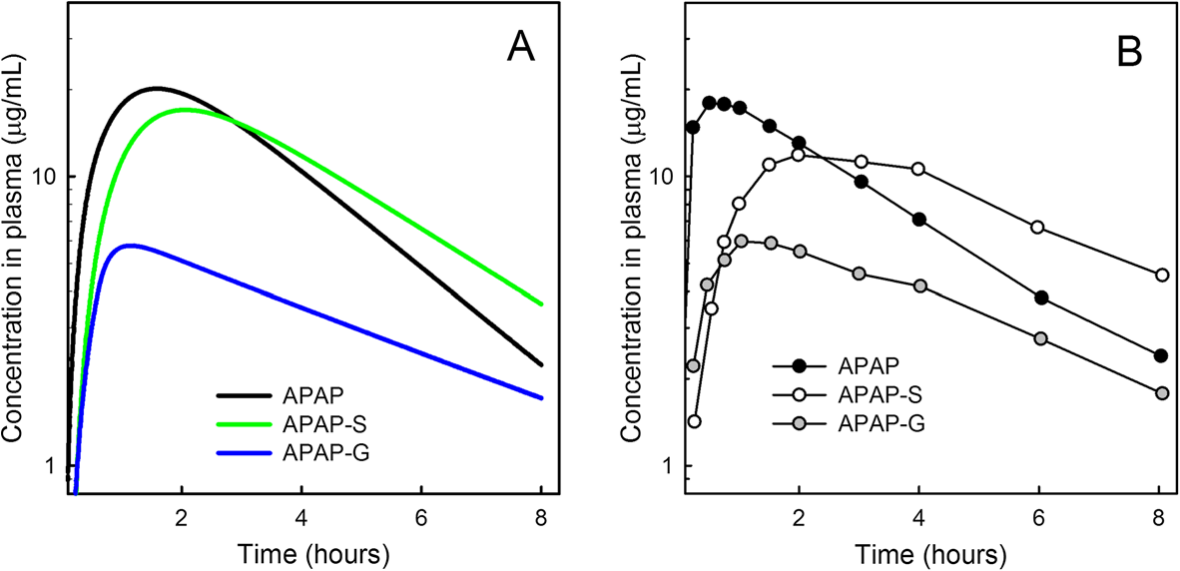
**Times courses in the plasma.** Panel **A** shows model calculations of the time courses of APAP, APAP-S, and APAP-G in the plasma after an APAP dose of 20mg/kg. Panel **B** shows the values measured in the plasma redrawn from Prescott et al. [4].

We modeled an oral dose of APAP by setting an initial value in the gut compartment. From the gut compartment, the dose first enters the liver where some of it is metabolized and conjugated, and the rest enters the general circulation from where it is taken up by liver and tissues or excreted in the urine. The profile of APAP and its glucoronidate and sulfate conjugates in the plasma after a 20 mg/kg dose were studied in [4] and are shown in Figure 2B, and the results computed by our model for the same dose are shown in Figure 2A. The match to the experimental data is excellent.

**Figure 3:**
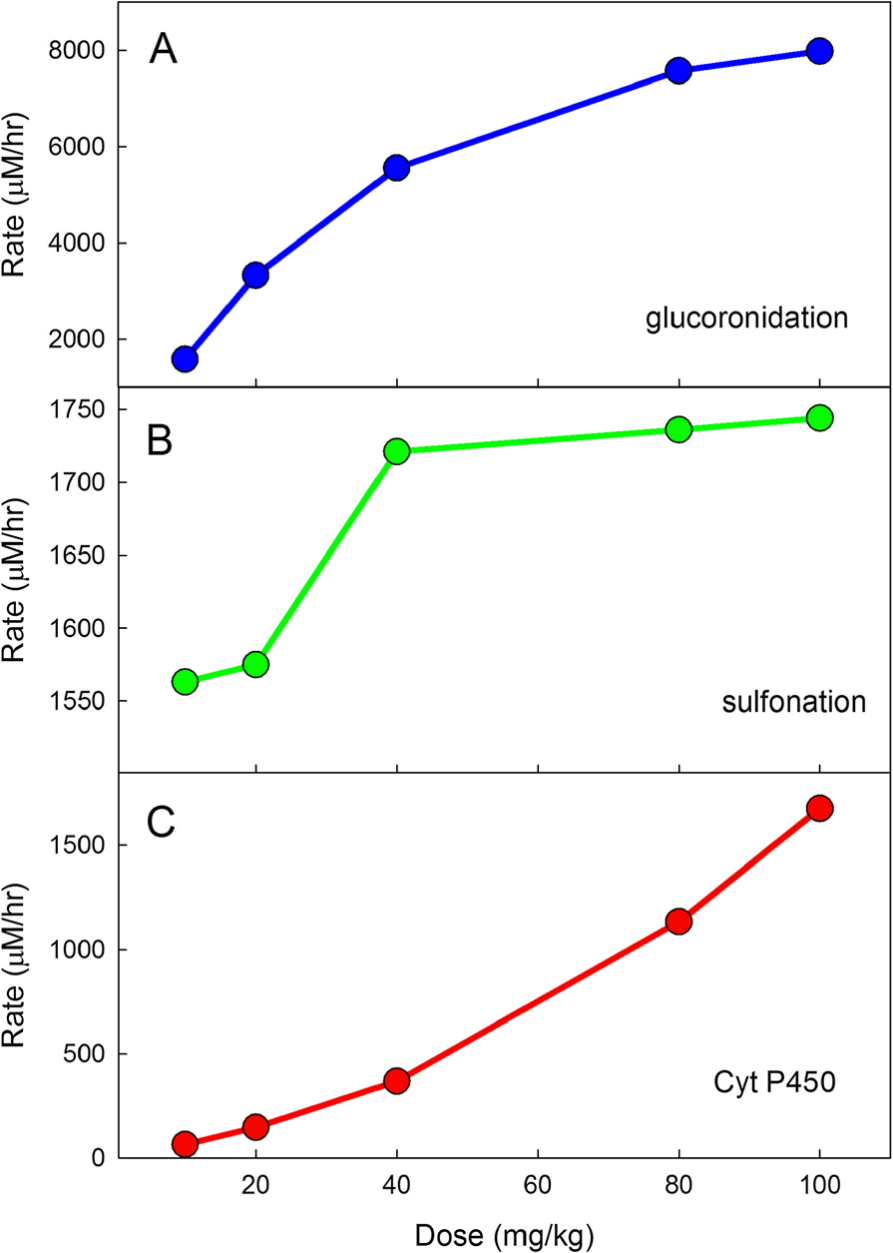
**Liver reaction velocities as function of dose.** Panel **A** shows the sum of the rates of the glucoronidation reactions in the liver 0.5 hours after the dose for a range of doses. The normal dose is 20 mg/kg assuming a 60 kg individual. Similarly, Panels **B** and **C** show the rates of the sulfation reaction and the sum of the P450 reactions, respectively. The sulfation reaction saturates at relatively modest doses.

**Figure 4:**
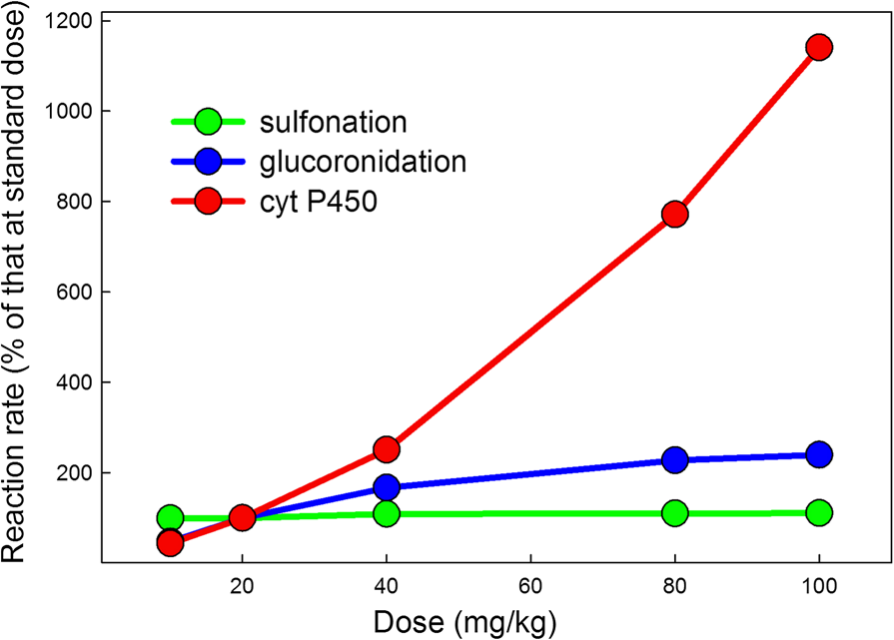
**Liver reaction velocities as percent of velocities for a therapeutic dose.** The velocities of the glucoronidation, sulfation, and P450 reactions are shown as a percentage of the velocities for a normal dose of 20 mg/kg at 0.5 hours after the dose. Note the steep rise of the P450 reactions as the dose increases.

**Figure 5:**
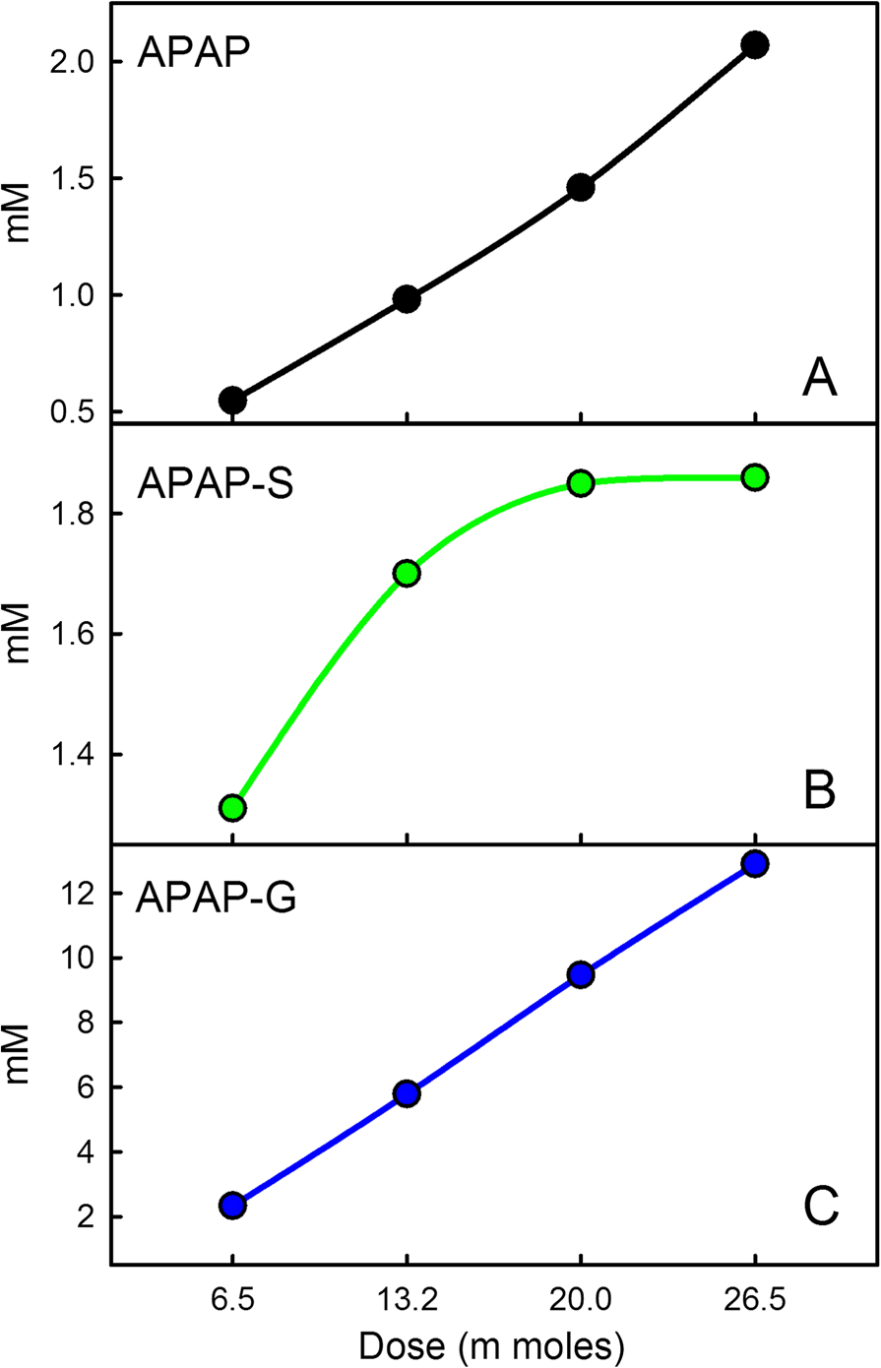
**Accumulation of metabolites in the urine.** Panels **A**, **B**, and **C** show the accumulation of APAP, APAP-S, and APAP-G, respectively, in the urine over a 24 hour period as a function of APAP dose. The results correspond well to those reported in [7]. We give the dose in moles for easy comparison to the experimental data.

It is known that high doses of APAP are toxic for two reasons. First, the sulfonation reaction saturates and that allows more NAPQI to accumulate ( [7,24]) and second, the increased amount of NAPQI exhausts the liver stores of reduced glutathione (GSH) [6] as well as the liver’s capacity to synthesize new GSH. In Figure 3, we show model computations of the rates of the glucoronidation reaction, the sulfation reaction and the cytochrome P450 reaction in the liver at 0.5 hours after the dose for a range of doses. The sulfonation reaction saturates at relatively modest doses, but the rates of the glucoronidation reaction and the rate of formation of NAPQI by the P450 reaction increase monotonically with dose. The dramatic increase in the synthesis of NAPQI is seen in Figure 4 where we plot the velocities as a percentage of their value relative to those computed at a standard dose. In Figure 5 we show the millimoles of APAP, APAP-S, and APAP-G that accumulate in the urine over a 24-hour period in the model for a range of doses. These elimination rates correspond well with the data in [7].

**Figure 6:**
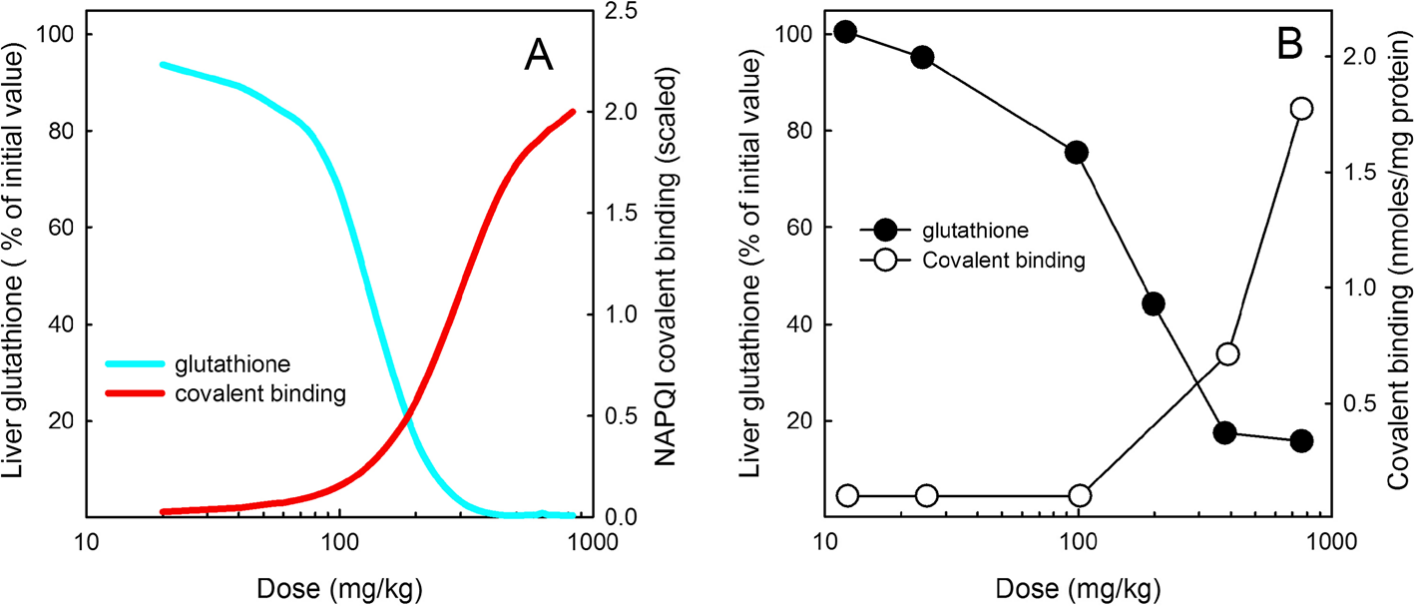
**Glutathione depletion and covalent binding.** Panel **A** shows model results. The blue curve shows the liver GSH concentration as a percentage of normal (left scale) 2 hours after the a dose of APAP as a function of dose size. The red curve shows the concentration of covalent binding of NAPQI (right scale) scaled to equal 2 at extremely high doses for easy comparison with the results of Mitchell et al. [6]. Panel **B** shows the comparable experimental results redrawn from [6] for the same two quantities.

Mitchell et al. [6] measured the level of GSH depletion in the liver (of mice), and the amount of covalent binding of radiolabeled APAP metabolites (later identified as NAPQI) in the liver after a wide range of doses of APAP. They showed that doses above 400 mg/kg caused an almost complete exhaustion of GSH levels in the liver and a sharp rise in the amount of covalent binding (Figure 6B). We used our model for APAP metabolism, integrated with our model for GSH metabolism [9] to compute the concentration of hepatic GSH and the amount of covalent binding of NAPQI after various doses of APAP. These model results are shown in Figure 6A and show a close similarity to the experimental results of [6] shown in Figure 6B. Mitchell et al. [6] reported covalent binding in units of nanomoles per milligram protein, whereas in our program we calculate NAPQI covalent binding in units of molarity. In Figure 6B we scale our units so they are “2” at dose of 833 mg/kg so they correspond numerically to the values given by Mitchell et al. [6] and are more easily compared.

### Effect of chronic dosing

The recommended therapeutic dose of APAP is 1000 mg not more than four times per day ( [33,34]). In Australia and new Zealand, the recommended dose is 500 to 1000 mg every four to six hours, not to exceed 4000 mg per day [1]. In the USA, the maximum dosage per day recommended by the manufacturer (MacNeil Consumer Healthcare) was reduced from 4000 mg (eight 500 mg pills) to 3000 mg (six 500 mg pills) in 2011 [35].

Although high doses of APAP are well known to be associated with increased risk of liver failure, chronic exposure to standard therapeutic doses is also not without risk. Forget et al. [36] report on two cases of acute liver failure after 3 and 10 days of therapeutic APAP treatment, respectively, in patients with liver steatosis. Nuttall et al. [37] studied the effect of chronic ingestion of therapeutic doses of APAP (1 gram, 4 times per day, for 2 weeks) on serum antioxidant capacity, and found a gradual and progressive decline to a level about 85% of control value. Part of the antioxidant capacity of serum is due to the presence of GSH and the reduced capacity could be associated with a general reduction in GSH due to its conjugation with NAPQI. Watkins et al. [38] studied the effect of chronic APAP ingestion (using the same protocol of 1000 mg every 6 hours for 14 days) on liver damage as measured by elevation of serum alanine aminotransferase (ALT). They found ALT elevations of up to 8 times the upper limit of normal in 8% of participants, and three times the upper limit of normal in 39% of participants. This study was stopped early due to the frequency and magnitude of the elevation in ALT in the treatment group relative to controls, although none of the participants expressed symptoms of liver disease. In a prospective study, Sabate et al. [39] estimated the incidence of acute liver injury due to therapeutic dosages of APAP to be about 10 per million user-years. These studies show that chronic usage of APAP at recommended therapeutic levels probably does mild liver damage and may be associated with a reduction in GSH levels that compromise antioxidant defense capacity.

**Figure 7:**
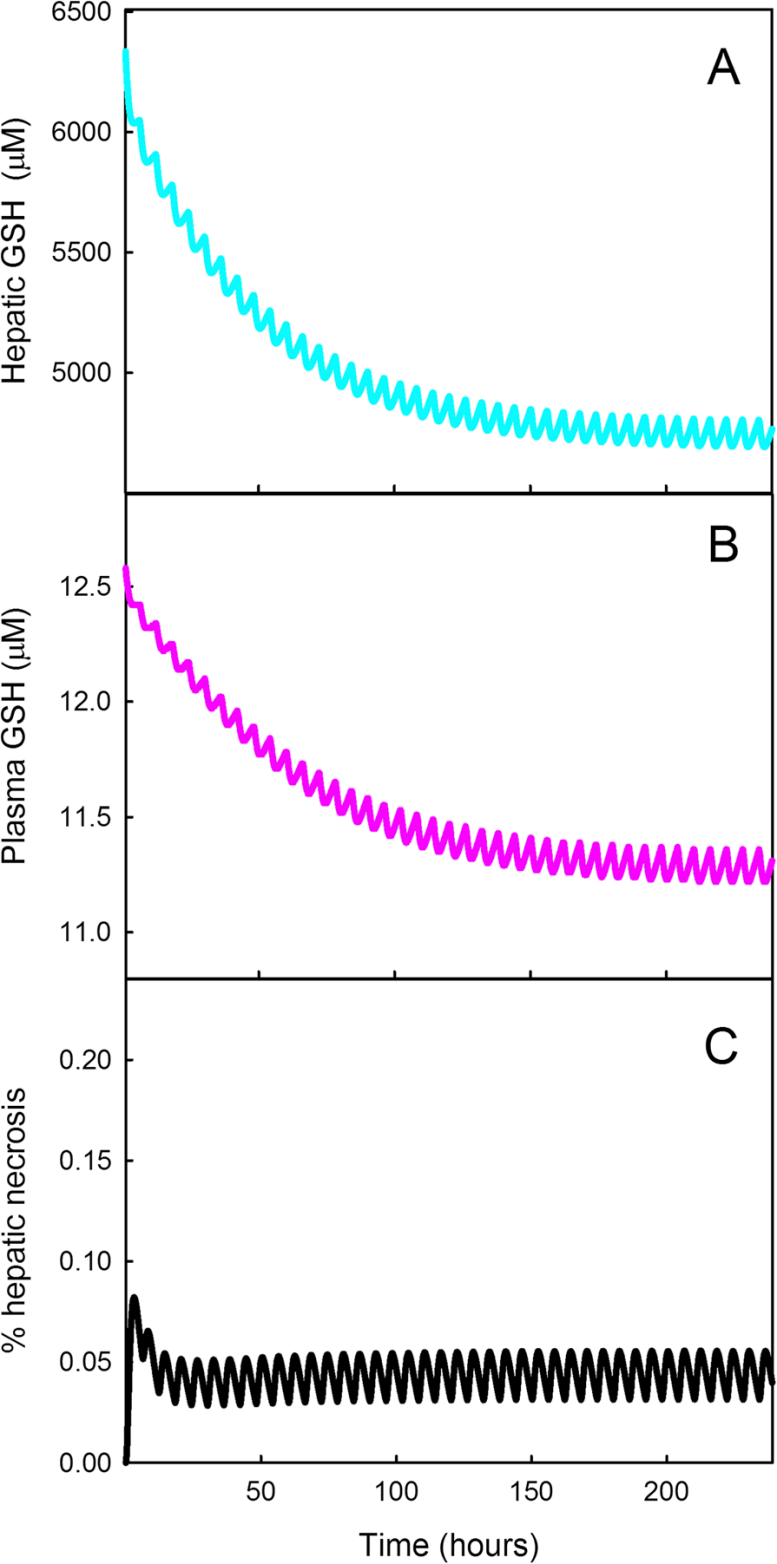
**Glutathione depletion and hepatic necrosis under chronic therapeutic dosing.** Panels **A**, **B**, and **C** show the model computations liver GSH, plasma GSH, and liver necrosis as a result of therapeutic dosing of APAP(1 gram every 6 hours) for 10 days. The curves reach their new steady states after about 150 hours. The curves oscillate because of the period dosing of APAP, the resynthesis of GSH in the liver, and regeneration of cells in the liver.

We used our model to study the effect of repeated doses of APAP on liver and serum GSH levels, NAPQI binding and estimated liver damage. We computed the effect of a 1000 mg dose every 6 hours for a period of 10 days. In our simulations (Figure 7, panels A and B) liver GSH declines to 70% of normal and plasma GSH declines to 88% of normal. These new dynamic steady states are achieved after about 150 hours. In comparison, Nuttall et al. [37] found that antioxidant capacity of serum continued to decline for 2 weeks and declined to 85% of normal. In Figure 7C we show an estimate of liver damage done by these chronic doses. The estimate of liver necrosis is rather small, less that 0.05% damage, and this may be sufficient to account for the elevation of ALT observed by Watkins et al. [38], and the absence of symptoms of liver disease after chronic usage. The GSH curves oscillate because of the discrete dosing every six hours and the regeneration of GSH in the liver. The liver necrosis curve oscillates because cells that die during a dose are replaced by regenerated cells; we take the regeneration rate from [10]. Our model simulations suggest that chronic usage of APAP at recommended therapeutic levels probably does mild liver damage and may be associated with a reduction in GSH levels that compromise antioxidant defense capacity.

### Effect of drugs that affect P450 activity

The toxicity of acetaminophen is due to the action of several P-450 cytochromes (CYPs) that catalyze the synthesis of NAPQI from APAP. The activity of these enzymes is enhanced by a variety of chemicals, including caffeine [40,41] and anticonvulsant drugs [42], and it is well known that co-ingestion of these drugs with APAP can greatly enhance the toxicity of APAP.

A relationship between the consumption of ethanol and the toxicity of APAP has also long been known [43]. In rats and mice, chronic exposure to alcohol causes an increased expression of CYP-2E1 and increases the activity of the enzyme 5- to 7-fold [44,45]. In humans the effect is much less dramatic, and alcohol consumption causes a transient two-fold induction of CYP-2E1 ( [43,46]). The role of alcohol in enhancing the toxic effects of APAP is variable and acute alcohol doses may have different effects on P-450 induction than chronic exposure to alcohol [43]. Exposure of cultured human hepatocytes to alcohol increased the expression of CYP-2E1 and CYP-3A3/4 up to 6-fold, but the effect appeared to be individually variable [47].

**Figure 8:**
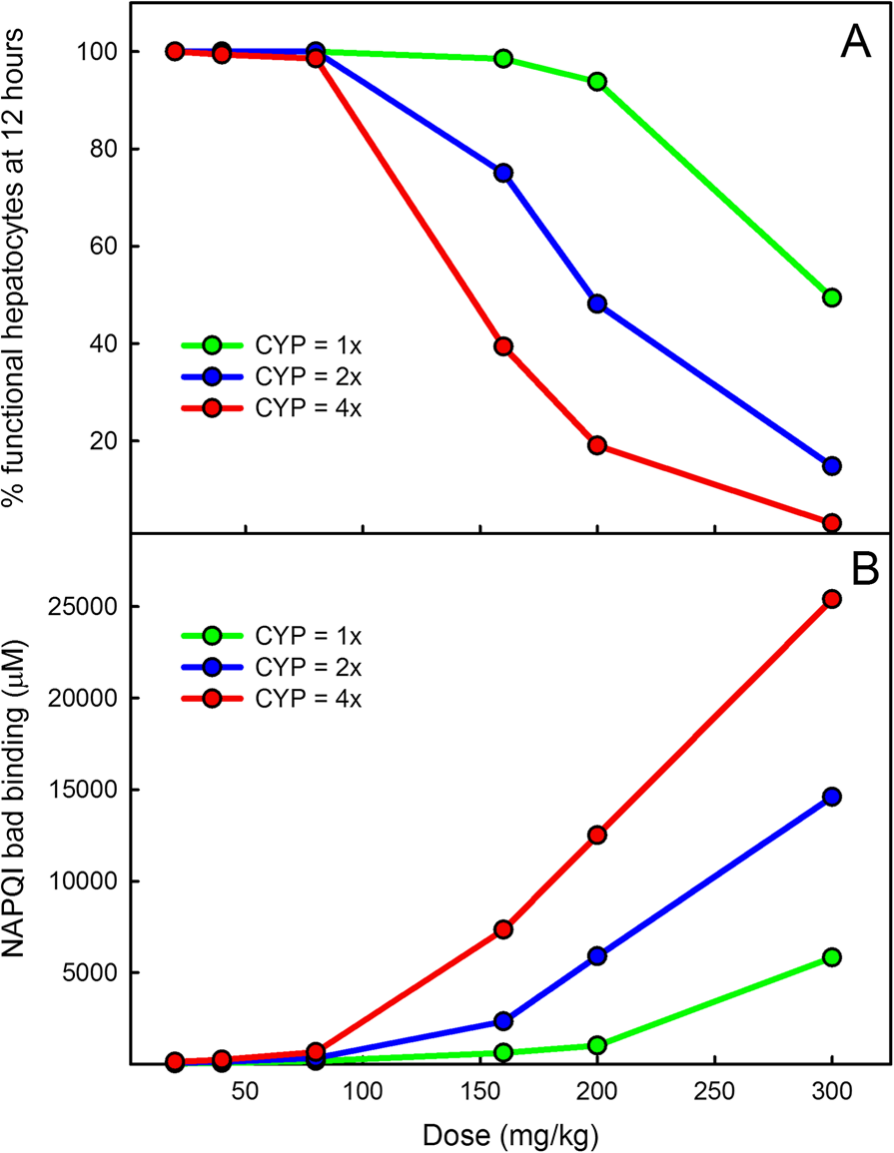
**The effects of APAP dose and CYP activity on covalent binding and necrosis.** Panel **A**: Model computations show the effect of P450 activity on liver necrosis at 12 hours after APAP doses of various sizes (normal, green; twice normal, blue; four times normal, red). Liver failure is thought to occur when the percent of functional hepatocytes falls below 30% [10]. Panel **B**: Model computations of the effect of of P450 activity on the covalent binding of NAPQI at 12 hours after APAP doses of various sizes (normal, green; twice normal, blue; four times normal, red). For relatively modest doses, P450 activity has little effect in both cases. However, the effect of P450 activity is dramatic for high doses.

We used our model to study the effect of increased activity of the P450 enzymes on the level of NAPQI covalent binding and the predicted associated level of hepatic cell necrosis. In Figure 8 we show the effect of two-fold and four-fold increases in P450 activity on NAPQI covalent binding at different doses of APAP. Covalent binding increases with APAP dose and with P-450 activity and the increase is non-linear. Doses of 2 to 4 times the therapeutic dose have only small effects, but the effect increases rapidly with doses above 8 times the normal therapeutic dose if P-450 activity is elevated.

Prescott [43] has suggested that increased APAP toxicity in the presence of alcohol may occur only when the liver is already compromised by other factors. Our finding that there is only a small increase increase in covalent binding after a therapeutic dose, even with a four-fold increase in CYP P-450 activity supports this idea.

### Effects of polymorphisms in glucoronosyl transferases

There are two reasons to expect that the glucoronosyl tranferase enzymes may be crucial for preventing liver damage. First, as we have shown above in Figures 4 and 5, the sulfation reaction saturates at fairly low APAP doses because of the low concentration of PAPS. Second, although the conjugation of the toxic intermediate NAPQI by glutathione is an important protective mechanism, it occurs after the production of NAPQI, while glucoronidation removes APAP before the production of NAPQI. Furthermore, a large number of genetic variants have been described in the UGT genes that are due to mutations in both the coding and regulatory regions of the genes [48-53]. These genetic variants are common and can have profound effects on the APAP glucoronidation capacity of the liver. For instance, Fisher et al. [48] found up to 7-fold differences in the rates of APAP glucoronidation in a sample of 20 human livers, and Court et al. [54] found 15-fold inter-individual variability in APAP glucoronidation rates in liver microsomal fractions.

**Figure 9:**
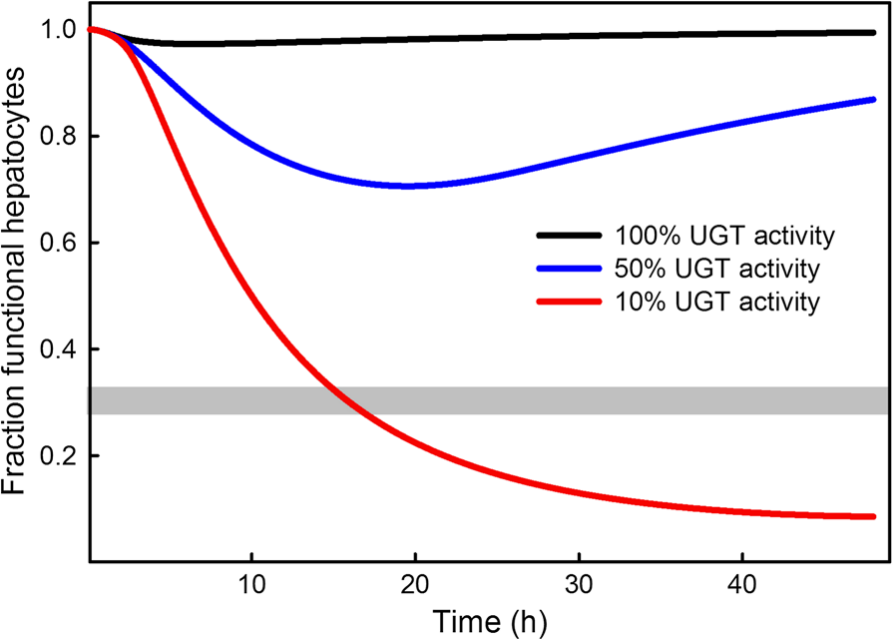
**Polymorphisms in glucoronosyl transferases affect liver damage.** Many polymorphisms in glucoronosyl tranferase enzymes reduce their activity by 50% or more (see text). The black, blue, and red curves show model calculations of the time courses of the percentage of functional hepatocytes in response to a 10 g overdose if the *V*_*max *_for the four glucoronosyl tranferases are normal (as given in Methods), 50% of normal, or 10% of normal, respectively. The activity of the glucoronosyl tranferases has a dramatic effect on liver damage. With normal parameter values (black curve) there is almost no hepatocyte death. However, at the 10% level (red curve), the number of functional hepatocytes decreases well below the 30% level thought to be the threshold for liver failure [10].

We used our model to test the importance of glucoronidation and it’s sensitivity to the activity of the glucoronosyl tranferase enzymes by computing the amount of liver damage resulting from a moderate overdose (10 g) with different choices for the *V*_*max*_ values of the glucoronosyl tranferases. With the normal values (given in Methods) of *V*_*max*_ for the four glucoronosyl tranferases, there is almost no liver damage (the black curve in Figure 9). When the *V*_*max *_values are set to 50% of their normal values, the number of functional hepatocytes decreases to 75% of normal after 20 hours (the blue curve). And, when the *V*_*max*_ values are set to 10% of their normal values, the number of functional hepatocytes decreases to 10% of normal after 40 hours (the red curve), well below the grey bar marking 30% remaining hepatocytes, which is thought to be the threshold for liver failure [10].

### Glutathione depletion and N-acetylcysteine rescue

**Figure 10:**
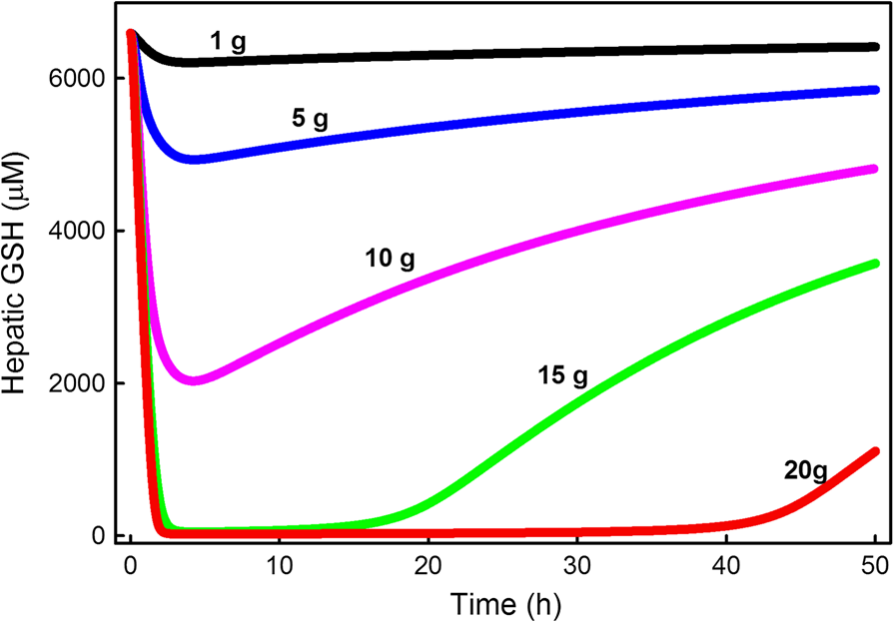
**Liver GSH depletion and recovery.** The black, blue, magenta, green, and red curves show liver GSH depletion and recovery after a therapeutic dose (1 g) of APAP, and after 5 g, 10 g, 15g, and 20 g doses, respectively. Liver GSH is almost completely depleted between 2 hours and 10 hours after the 15 gram dose and after 48 hours liver GSH has recovered only to about 1/2 of normal. Even for the therapeutic dose, liver GSH has not completely recovered after 48 hours.

Since the purpose of NAC rescue is to replenish GSH in the liver, it is important to know the time course of GSH in reponse to various doses and how quickly it recovers. Because our acetaminophen model is connected to our GSH model we can compute these time courses explicitly. In Figure 6 we showed that an overdose of APAP (corresponding to 200 mg/kg, or a 12 g dose for a 60 kg person) depletes liver GSH severely after 2 hours. Figure 10 shows the time-line of decline and recovery of hepatic GSH after a therapeutic dose (1 g), and after 5 g, 10 g, 15 g, and 20 g doses, respectively. The 20 g dose is borderline lethal without NAC rescue. The reduction in hepatic GSH after a therapeutic dose is minor, but a 15 g dose almost completely depletes hepatic GSH between 2 and 10 hours. For the 20 g dose, liver GSH does not start to recover until 40 hours after the dose. In all cases, including a therapeutic dose, it takes more than 48 hours for GSH to recover to its original steady-state. In the cases of 15 g and 20 g doses the liver concentration of GSH stays very low for an extended period of time. This does not mean that conjugation of NAPQI is not taking place. NAPQI is being conjugated at the rate at which new GSH is being synthesized, which keeps the concentration of GSH from rising.

**Figure 11:**
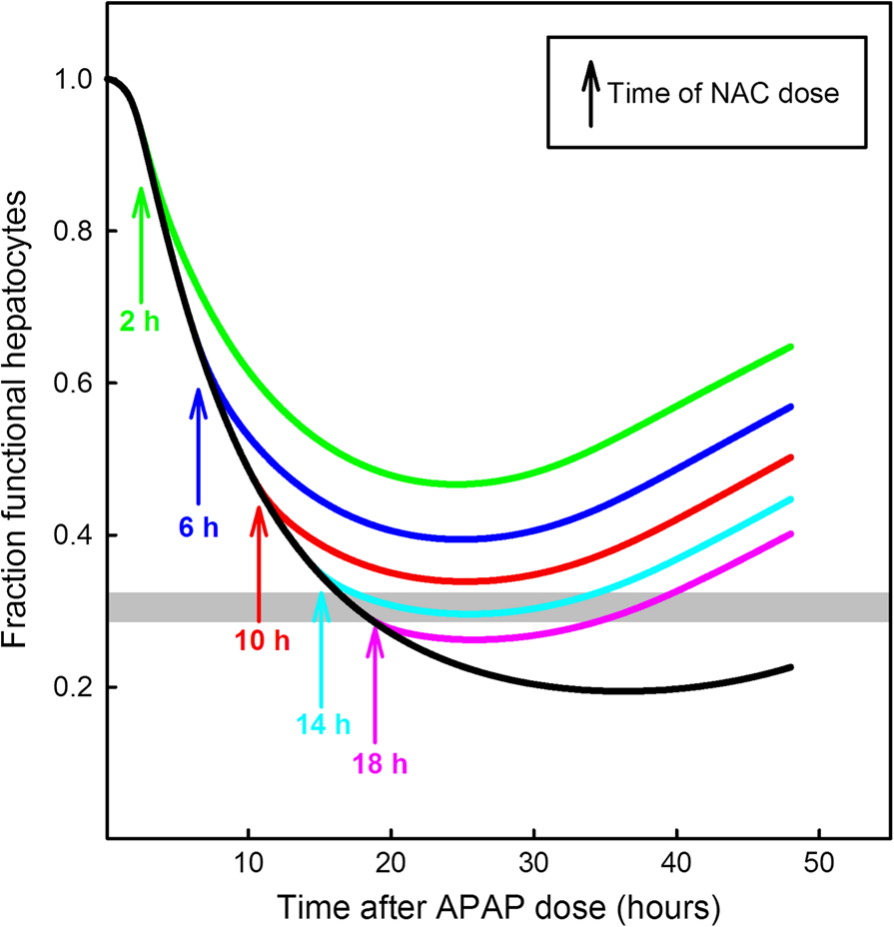
**Time course of the percent functional hepatocytes after a 22 g overdose and different NAC rescue times.** The black curve shows model computations of the percentage of function hepatocytes after an 22 g dose of APAP. The curve decreases well below the gray bar at the 30% level below which liver failure usually occurs. The green curve shows the time course of functional hepatocytes with rescue by 36 mM NAC given continuously for one hour starting at 2 hours after the overdose. The green curve stays well above the 30% threshold. The blue, red, cyan, and magenta curves show the time courses of functional hepatocytes if the rescue dose is given at 6, 10, 14, 18 hours respectively. The curves show clearly the importance of early rescue.

The literature on NAC rescue for acetaminophen overdose makes it clear that early rescue is important [10,55,56]. We used the model to investigate the effect of the timing of N-acetylcysteine (NAC) rescue. We assume a 22 g dose of APAP followed by an infusion of 36 mM NAC (the standard rescue dose [1]) over a 1 hour period starting at various times after the APAP dose. The black curve in Figure 11 shows the time course of the percentage of functional hepatocytes after the 22 g dose. It decreases well below the shaded horizontal bar that represents 30% hepatocytes left, the level below which liver failure is thought to occur [10]. The green curve shows the time course of the percentage of functional hepatocytes if the NAC rescue is performed at 2 hours after the dose was ingested; the curve stays well above the 30% level. The blue, red, cyan, and magenta curves show the time courses of the percent functional hepatocytes if the rescue dose is given at 6, 10, 14, 18 hours respectively. The cyan curve is borderline for liver failure and the magenta curve is well below the 30% bar. Notice that in all cases the percentage of functional hepatocytes continues to decrease for some time after the NAC rescue. These curves show clearly the importance of early NAC dosing for saving patients. Of course, with a smaller overdose, the curves would be higher and with a larger overdose the curves will be lower.

**Figure 12:**
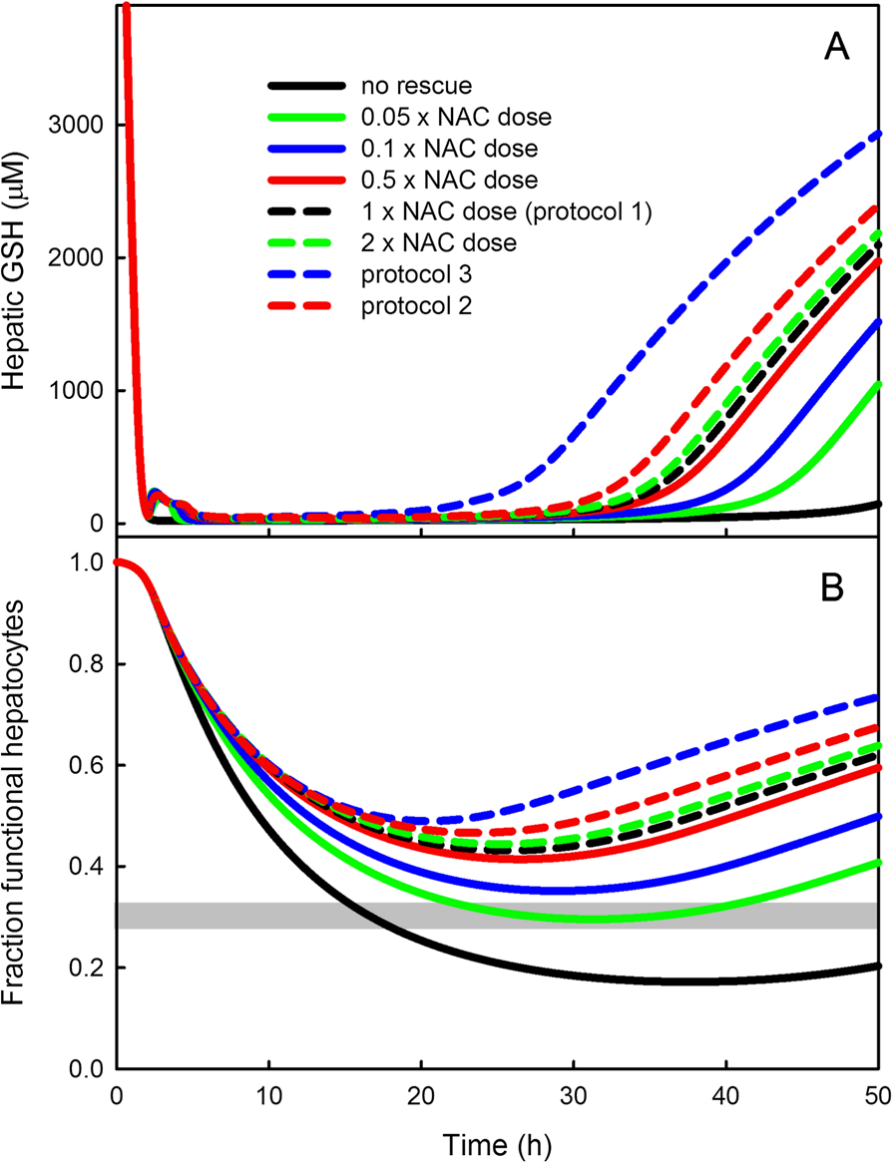
**The effects of different NAC doses and rescue protocols on liver GSH and functional hepatocytes.** Panel **A** shows time courses liver GSH concentrations and Panel **B** shows time courses of functional hepatocytes. For all simulations the APAP dose was 22g, which is lethal without NAC rescue. The black curves show the time course of liver GSH and functional hepatocytes with no NAC rescue. The dashed black curves show the effects of rescue with a dose of 36mM NAC given over a one hour period starting two hours after the APAP dose (protocol 1). If we rescue over one hour with twice as much NAC (green dashed curves) or half as much NAC (solid red curves) the results are very similar. However, rescue with 1/10 (solid blue curves) and 1/20 (solid green curves) the normal NAC dose over a one hour period starting at 2 hours show much poorer rescue; at 1/20 the patient’s hepatocytes decline to 30% and survival is in doubt. The dashed red curves and the blue dashed curves show the time courses of liver GSH and functional hepatocytes corresponding to the dosing protocols 2 and 3 as described in the text. In these protocols, the NAC dose is spread out over time. Protocol 3 is better than protocol 2, which is better than protocol 1.

We used the model to test how sensitive the model is to the size of the NAC rescue dose. In Figure 12, the black curves show the GSH concentration in the liver (Panel A) and the fraction of functional hepatocytes as a function of time (Panel B) after a 22 g dose of APAP. The dashed black curves show the responses of GSH and fractional hepatocytes to rescue by the standard dose of NAC (36 mM) given over a 1 hour time period starting at two hours after the APAP dose. We refer to this rescue as protocol 1. With this early rescue dose, the patient likely survives because the dashed black curve in Panel B stays well above the 30% level for functional hepatocytes, which is thought to indicate liver failure. Other curves show the responses to giving different amounts of NAC over this one hour period. Doubling the amount of NAC (dashed green) has very little effect on rescue and neither does halving the amount of NAC (solid red). However, 1/10 the normal NAC rescue (solid blue) and 1/20 the normal NAC rescue (solid green) substantially delay the time of GSH rebound and decrease substantially the curves showing the time course of functional hepatocytes. At 1/20 the normal rescue dose, the hepatocyte curve descends to the 30% level so the survival of the patient is unclear.

We also simulated the effect on hepatic GSH level and functional hepatocytes of two other dosing protocols. In protocol 2, we give 3.6 mM of NAC per hour for 10 hours beginning 2 hours after the 22 g APAP dose. Thus, the total amount of NAC infused was identical to the standard amount given in a 1 hour-infusion (protocol 1). Our simulations (red dashed curve in Figure 12) show that protocol 2 does better than protocol 1: the red dashed curves are higher than the black dashed curves. Finally, we simulated the NAC dosing protocol recommended in [1], which we refer to as protocol 3. This protocol consists of infusing half the 36 mM NAC dose over a 1-hour period starting at 2 hours after the APAP dose, followed by 1/6 of the dose over the next 4-hour period and then 1/3 of the dose over an additional 16 hours. The blue dashed curves in Figure 12 show the time course of GSH (Panel A) and the time course of functional hepatocytes (Panel B) for protocol 3. Protocol 3 is substantially better than protocol 2, which is better than protocol 1.

### Predicted death or recovery

**Figure 13:**
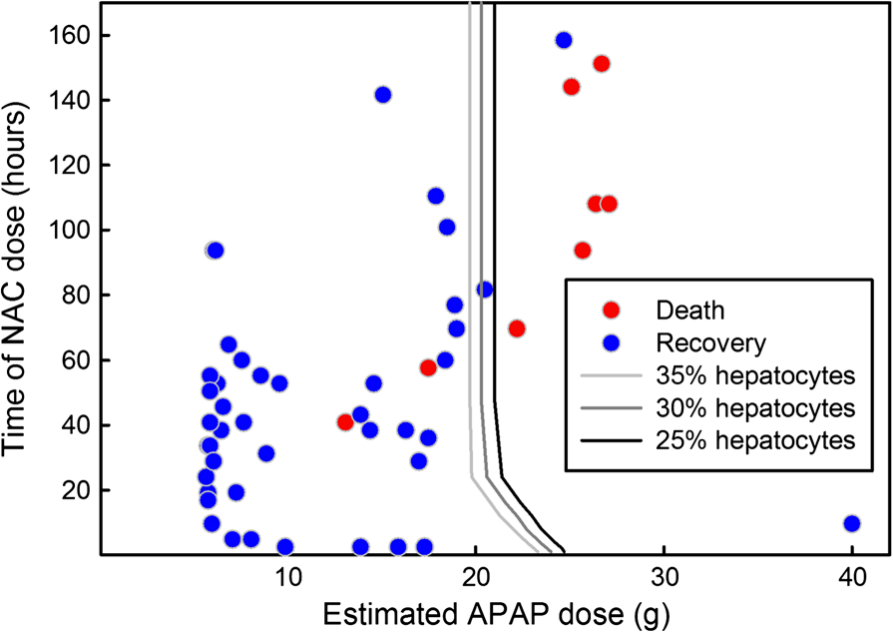
**Predicted death or recovery.** Each dot represents one patient (who did not receive a liver transplant) and it is plotted so that the x coordinate is the dosage estimated by Remien et al. [10] and the y coordinate is the time since the dose estimated by Remien et al. [10]. Death or recovery is indicated by red or blue, respectively. We indicate the curve of hepatocyte depletion to 35% of normal (light grey), 30% of normal (medium grey), and 25% of normal (dark grey). If we use the 30% curve as the boundary between predicted death and predicted recovery, then we incorrectly predict only 3 deaths and 2 recoveries.

Remien *et al*[10] developed a model that allowed them to use patient values of plasma indicators of liver damage (plasma levels of aspartate aminotransferase, alanine aminotransferase, and the international normalized ratio of prothrombin time) to estimate the APAP dose and the time of dose. Their model then calculates a prediction of death or recovery using using 30% remaining hepatocytes as the boundary between death and recovery. They compared their model with the patient records of 53 patients at the University of Utah Medical Center and their predictions of death or recovery were quite accurate. To compare the predictions of our model to the patient records, we used their predictions of size and timing of dose for each of the 53 patients. Then we computed using our model whether the functional hepatocytes ever declined below 30% in which case we predict death rather than recovery. Figure 13 shows the outcome for the 53 patients in the study of Remien et al. [10]; blue indicates recovery, red indicates death, and each dot is plotted so that the x-coordinate is the Remien-predicted dose and each y coordinate is the Remien-predicted time since dose when the patient appeared in the Emergency Department. In Figure 13, the medium grey line shows the coordinates that result in 30% remaining hepatocytes. Thus our model predicts recovery to the left of the medium gray line and death to the right of the medium gray line. For reference, the curves for 35% remaining hepatocytes (light grey) and 25% remaining hepatocytes (dark grey) are also shown. Our model predicts outcomes very well; only two of our predicted recoveries died and three of our predicted deaths survived.

## Discussion

We have created a whole body model of acetaminophen transport and metabolism that includes the details of the biochemical pathways of acetaminophen metabolism in the liver and peripheral tissues. The model was based as much as possible on parameters from the biochemical literature. When compared to experimental and clinical data on the accumulation of the byproducts of acetaminophen metabolism, APAP-S, APAP-G, and NAPQI-GSH, in the plasma and in the urine of humans, the model gives accurate predictions (see Figures 2, 3, 4 and 5).

We connected the whole body model of acetaminophen metabolism to our previously constructed model of glutathione metabolism [9] so that we could study the depletion of GSH after APAP doses of various sizes (Figure 6). We found that therapeutic doses decrease liver GSH by only modest amounts (10%), but that overdoses of 10 grams or more severely deplete liver GSH (Figure 6). In addition, chronic therapeutic doses do deplete liver GSH significantly (30%; Figure 7). Futhermore, it takes more than two days for the liver to synthesize enough GSH to bring concentrations back to normal (Figure 9). Our model results correspond well with measurements of plasma GSH after doses of various sizes (Figure 6).

Acetaminophen is toxic to hepatocytes because of the production of the intermediate, NAPQI, by cytochrome P450 enzymes. Thus it is not surprising that compounds that increase the activity of the P450 enzymes, such as caffeine [40,41] and anticonvulsant drugs [42] also make APAP more hepatotoxic. There is also a connection between alcohol consumption and APAP hepatotoxicity [43], and again the presumed mechanism is an increase in activity of one or more P450 enzymes [46,47]. We show in Figure 8 that the effect of increasing the activity of the P450 enzymes is highly nonlinear. At low doses of APAP there is little effect while the hepatotoxicity increases rapidly at high doses.

In Section “Effects of polymorphisms in glucoronosyl transferases” we showed that polymorphisms in the glucoronosyl transferase enzymes can have a large effect on the amount of liver damage caused by moderate overdoses of APAP. Figure 9 shows that under normal circumstances, a 10 g dose does not cause much liver damage. However, if polymorphisms in the glucoronosyI transferases reduce the *V*_*max*_ values to 50% of normal then the number of hepatocytes drops to about 75% of normal after 20 hours. And, if polymorphisms in the glucoronosyI transferases reduce the *V*_*max*_ values to 10% of normal then the number of hepatocytes drops to about 10% of normal after 20 hours. Thus, liver damage is quite sensitive to polymorphisms in the glucoronosyl transferases and this probably explains some of the variation in patient response.

It is known that the ability to detoxify APAP varies greatly among different animal species [57]. In particular, cats are acutely sensitive to APAP hepatotoxicity because they do not glucoronidate APAP well in their livers [58]. Because our model includes the metabolic pathways in the liver, after some modifications, it can be used to study APAP metabolism and toxicity in other species, a task that we plan to take up in the future.

The standard antidote for APAP overdoses given in Emergency Departments is N-acetylcysteine (NAC) which is given to increase GSH production. Our model confirms the clinical observation that NAC rescue within 8 hours of overdose is usually successful in preventing liver failure (Figure 11). We take liver failure to be equivalent to less than 30% remaining hepatocytes as suggested in [10]. We then used the model to study different dosing strategies and found that modest differences result from different dosing strategies as long as they are started early enough (Figure 12).

An important check on our model was to compare it’s predictions on patient outcomes to the empirical data and modeling results presented in [10]. Both our model and theirs predict accurately death or recovery in the 53 patients studied by (Figure 13). Their model, which is much simpler than ours in that it does not contain detailed liver biochemistry, is sufficient for predicting patient outcomes. The purpose of our larger model is to provide a platform for experimentation with NAC dosing protocols, and with the effects of genetic polymorphisms, expression levels of enzymes, diet, the depletion of GSH, and the effects of environmental enzyme activators or inhibitors such as caffeine and alcohol.

## Competing interests

The authors declare that they have no competing interests.

## Authors’ contributions

RB and YC wrote the final code for the model. SL and CH did background research and wrote a preliminary version of the APAP metabolism code. The project was directed by MR and HFN who also wrote the manuscript. Thanks are due to Shira Rubin for discussions about acetaminophen toxicity in animals. All authors read and approved the final manuscript.
